# *Plasmodium falciparum* Produce Lower Infection Intensities in Local *versus* Foreign *Anopheles gambiae* Populations

**DOI:** 10.1371/journal.pone.0030849

**Published:** 2012-01-26

**Authors:** Caroline Harris, Isabelle Morlais, Thomas S. Churcher, Parfait Awono-Ambene, Louis Clement Gouagna, Roch K. Dabire, Didier Fontenille, Anna Cohuet

**Affiliations:** 1 Institut de Recherche pour le Développement, UMR 224 Maladies Infectieuses et Vecteurs: écologie, génétique, évolution et contrôle, Montpellier, France; 2 Laboratoire de Recherche sur le Paludisme, Institut de Recherche pour le Développement, IRD-OCEAC, Yaoundé, Cameroon; 3 Department of Infectious Disease Epidemiology, Faculty of Medicine, Imperial College London, London, United Kingdom; 4 Institut de Recherche en Sciences de la Santé, Bobo Dioulasso, Burkina Faso; Université Pierre et Marie Curie, France

## Abstract

Both *Plasmodium falciparum* and *Anopheles gambiae* show great diversity in Africa, in their own genetic makeup and population dynamics. The genetics of the individual mosquito and parasite are known to play a role in determining the outcome of infection in the vector, but whether differences in infection phenotype vary between populations remains to be investigated. Here we established two *A. gambiae s.s.* M molecular form colonies from Cameroon and Burkina Faso, representing a local and a foreign population for each of the geographical sites. Experimental infections of both colonies were conducted in Cameroon and Burkina Faso using local wild *P. falciparum*, giving a sympatric and allopatric vector-parasite combination in each site. Infection phenotype was determined in terms of oocyst prevalence and intensity for at least nine infections for each vector-parasite combination. Sympatric infections were found to produce 25% fewer oocysts per midgut than allopatric infections, while prevalence was not affected by local/foreign interactions. The reduction in oocyst numbers in sympatric couples may be the result of evolutionary processes where the mosquito populations have locally adapted to their parasite populations. Future research on vector-parasite interactions must take into account the geographic scale of adaptation revealed here by conducting experiments in natural sympatric populations to give epidemiologically meaningful results.

## Introduction

Malaria is caused by parasites of the genus *Plasmodium* which have a complex life cycle, obligatorily involving a vertebrate host and mosquito vector. On ingestion of an infectious blood meal, the parasite begins sporogonic development [1]. During this time the parasite causes damage to the mosquito [2] and triggers an immune response against it [3]–[4], however the true cost of infection remains unclear [5]. Theoretically, the outcome of infection could be that none of the parasites survive; the mosquito is overwhelmed and dies, or anything in-between. The persistence of malaria indicates that the in-between stage is commonly reached where parasites successfully infect at levels that do not kill the vector, at least not before transmission occurs [6]. Selective pressures exerted by the parasite on its host and vice versa are expected to drive evolution for each to maximize its fitness. In the vectorial stages, evolutionary forces will favor the parasites with the highest transmission [7], this could lead to adaptation between sympatric parasite and vector populations due to local adaptation [8]. The factors that regulate the success and level of infection in the malaria vectors are now under scrutiny but the role of natural population diversity and geographical variations remain overlooked.

*Plasmodium falciparum* is the most devastating human malaria parasite and is transmitted throughout tropical Africa and Asia and sporadically in Central and South America. In Africa its main vector is *Anopheles gambiae*, though other *Anopheles* species can play major roles on more local scales [9]. A degree of adaptation was suggested between geographically isolated populations of *A. gambiae* and *P. falciparum* when an *A. gambiae* colony was successfully selected for resistance to New World *P. falciparum* isolates but remained susceptible to those of African origin [10]. Different vector-parasite interactions may have evolved through adaptation in the African *A. gambiae* and *P. falciparum* allowing this parasite population to evade the mosquito immune response [11]. African and New World *P. falciparum* populations show moderate genetic divergence [12]–[13], which could drive the differences in their infectivity.

Within a wild mosquito population and sympatric wild parasite population, the outcome of infection depends on the combination of the individual mosquito and parasite genotypes [14]–[15], but whether differences in infectivity exist at the population level within Africa remains to be answered. A study on *Plasmodium vivax* in Mexico looked at infectivity of parasite populations to different mosquito species within an area of 100 m^2^. Parasite populations were characterized genetically and each found to produce higher infections in its sympatric mosquito species [16]. Malaria transmission can therefore segregate over very small geographical scales and could influence vector and parasite fitness when they interact with different local populations/species.

Differences in selective pressures (due to differences in transmission dynamics and virulence) infer inter-population divergence for selected loci [17]–[18]. Such genetic divergence between vector and parasite populations are a pre-requisite for local adaptation [19] and can lead to a mosaic effect of co-evolution/adaptation over space and/or time [20]–[21]. Co-evolution in some instances leads to local adaptation when an organism is fitter in its local environment [22]. Parasites are generally more likely to locally adapt to their hosts, rather than vice versa due to evolutionary advantages in terms of generation time, population size and rates of mutation and migration [19], [23]–[24], even if local mal-adaptation can occasionally occur [25]. In terms of malaria control, current efforts generally rely on destruction of either the parasite or mosquito [26]–[27]. It has been suggested that a novel way of approaching control could be to interfere with coadaptive processes between the human, mosquito and parasite [6]. In this study we compare the level of *P. falciparum* infection within sympatric and allopatric populations of *A. gambiae*, its natural vector, at two sites in Central and West Africa. Conclusions are drawn on the level of adaptation between the geographically isolated sympatric couples. To make results widely representative of nature wild parasite isolates and recently established mosquito colonies were used.

## Results

### Experimental infections

Nine experimental infections were carried out in Cameroon using local isolates of *P. falciparum* (hereafter named “PfCM”) to infect, in parallel, both the sympatric mosquito strain of *A. gambiae* colonized from the local population, “AgCM”, and the allopatric strain of *A. gambiae* originating from Burkina Faso, “AgBF” (Pf = *P. falciparum*, Ag = *A. gambiae*, CM = Cameroon, BF = Burkina Faso). All resulted in infections at the oocyst stage in mosquitoes of both strains with prevalences between 20–96%. 17 experimental infections were carried out in Burkina Faso, using local *P. falciparum* isolates, “PfBF”, to infect, in parallel both the sympatric mosquito strain; “AgBF” and allopatric strain; “AgCM”. 15 infections showed oocysts in both mosquito strains giving infection prevalence's between 8–100%. The number of mosquitoes fed on infectious blood and dissected on day 8 was between 36–140/infection for PfCM and 5–132/infection for PfBF. The mean number of oocysts per midgut ranged between 0.3–69.7 for PfCM and 0.2–21 for PfBF, Table 1.

**Table 1:** Infection summaries.

Parasite population	Tf/µl	Gams/µl	Mosquito strain	# of midguts dissected	Oocyst range	Oocysts/Midgut Arithmetic mean #	Oocysts/Midgut Geometric Mean	Prevalence (%)
**Pf CM**	240	257	Ag BF	94	0–146	41.6 (48.3)	18.9	86
			Ag CM*	140	0–117	44.9 (47.2)	30	95
	4320	370	Ag BF	58	0–254	69.7 (77.8)	36.1	90
			Ag CM*	112	0–131	31.6 (35.4)	17	89
	1110	107	Ag BF	53	0–152	44.7 (46.4)	26	96
			Ag CM*	108	0–98	24.5 (26.2)	14.3	94
	120	98	Ag BF	46	0–37	7.2 (9.7)	3.8	74
			Ag CM*	86	0–37	6.5 (8.1)	3.9	80
	30	68	Ag BF	120	0–29	3.9 (5.4)	2.6	73
			Ag CM*	128	0–16	4.3 (5.3)	3.2	82
	30	44	Ag BF	120	0–38	7.0 (9.8)	4	71
			Ag CM*	99	0–25	3.4 (5.3)	2.3	64
	690	23	Ag BF	81	0–17	2.5 (4.6)	1.9	54
			Ag CM*	82	0–13	2.4 (3.6)	1.9	67
	300	44	Ag BF	92	0–6	0.8 (2.4)	1.2	33
			Ag CM*	131	0–10	1.7 (2.9)	1.6	59
	600	21	Ag BF	66	0–4	0.3 (1.5)	1.1	20
			Ag CM*	36	0–2	0.3 (1.4)	1.1	22
**Pf BF**	180	54	Ag BF*	115	0–61	20.0 (23.7)	11.1	84
			Ag CM	46	0–45	18.2 (19.5)	11.4	93
	4920	66	Ag BF*	132	0–59	16.5 (18.9)	10	87
			Ag CM	90	0–52	17.3 (20.0)	11.4	87
	1045	84	Ag BF*	22	0–37	11.6 (17.1)	5.6	68
			Ag CM	91	0–61	20.2 (22.1)	15.1	91
	2340	336	Ag BF*	64	0–58	11.5 (14.7)	5.2	78
			Ag CM	15	0–63	16.9 (18.1)	9.8	93
	-	150	Ag BF*	73	0–60	9.4 (16.8)	3.6	56
			Ag CM	66	0–51	5.4 (11.8)	2.2	45
	480	30	Ag BF*	75	0–36	9.1 (10.5)	5.9	88
			Ag CM	93	0–70	16.7 (17.7)	12.6	95
	3060	48	Ag BF*	39	0–43	8.3 (11.6)	4.5	72
			Ag CM	34	0–33	11.4 (12.5)	7.5	91
	2000	72	Ag BF*	5	2–6	4.2	4	100
			Ag CM	24	0–60	21.0 (24.0)	17	88
	-	48	Ag BF*	45	0–19	3.4 (4.7)	2.3	73
			Ag CM	23	0–32	7.6 (9.2)	4.4	83
	1140	48	Ag BF*	24	0–8	2.5 (4.6)	2	54
			Ag CM	86	0–45	9.1 (12.0)	4.4	76
	-	30	Ag BF*	73	0–6	1.5 (2.5)	1.6	62
			Ag CM	119	0–18	3.9 (5.2)	2.8	75
	1200	66	Ag BF*	36	0–3	0.4 (2.1)	1.1	19
			Ag CM	26	0–5	0.2 (3.0)	1.1	8
	-	48	Ag BF*	56	0–4	0.4 (1.8)	1.1	21
			Ag CM	23	0–7	1.2 (2.2)	1.4	57
	7380	78	Ag BF*	62	0–3	0.3 (1.6)	1.2	16
			Ag CM	26	0–2	0.5 (1.3)	1.1	35
	-	36	Ag BF*	102	0–5	0.3 (1.4)	1	18
			Ag CM	93	0–5	0.7 (2.3)	1.2	30

Ordered according to arithmetic mean oocysts/midgut in sympatric combinations, denoted by a *. # arithmetic mean of infected mosquitoes only in brackets. Tf: trophozoites, Gams: gametocytes.

### Blood meal and wing size differences between mosquito strains

Blood meal size is expected to influence the number of parasites ingested and subsequently, the level of infection [28]–[30], while the size of mosquitoes is indicative of fitness [31] which could also influence susceptibility to infection [28], [32]. To estimate these potential biases, blood meal sizes and wing sizes were compared between mosquito strains. Blood meal sizes were determined in 127 AgCM and 138 AgBF females across 3 replicate feeds. The mean quantity of hematin measured was 17.49 µg/mosquito (SD = 6.58) for AgCM and 17.32 µg/mosquito (SD = 7.97) for AgBF. The Mann-Whitney test showed similar blood meal sizes in the 2 colonies in each separate replicate and when pooled (*p*>0.05). Wing sizes were measured for 50 AgCM and 48 AgVK females, giving means of 2.90 mm (SD = 0.11) for AgCM and 2.94 mm (SD = 0.16) for AgBF and again revealed no significant differences (*p*>0.05).

### Parasite population differentiation

The level of genetic diversity within a population is expected to drive adaptation at different rates [33]. We tested the genetic diversity within and divergence between the two parasite populations; PfBF and PfCM for two MSP (merozoite surface protein) loci. For the MSP1 K locus; 13 different alleles were detected from successfully genotyping 45 parasite positive blood isolates from Cameroon and 38 from Burkina Faso. For the MSP2 FC locus; 35 different alleles were present, from successfully genotyping 50 parasite positive blood samples from Cameroon and 35 from Burkina Faso. In Cameroon 83% of infections had multiple alleles present indicating mixed infections and 71% in Burkina Faso. Gene diversity, as determined by Nei 1987 [34], was found to be very high, with effective heterozygosity index's ranging from 0.8876–0.9112, depending on population and locus, showing that the two parasite populations are highly polymorphic. Analysis of MOlecular Variance (AMOVA) [35] showed that the two parasite populations are genetically indistinguishable at the MSP1 K locus. At the MSP2 FC locus, 1.38% of the variation is explained by between population differences (*p*<0.05, Fst = 0.01385) indicating low but significant divergence between the two populations.

### Differences in oocyst prevalence

The difference in the prevalence of mosquitoes infected by sympatric vs. allopatric parasite-vector combinations showed no significant differences (odds ratio of infection in the sympatric relative to the allopatric combination of 1.06, 95% CI: 0.88–1.26, for both parasite populations combined), as shown in Table 2. In analyzing each parasite population separately it shows that, in terms of prevalence, PfCM is more infective to its sympatric vector, while PfBF is more infective to its allopatric vector. This means that AgCM is infected significantly more often than AgBF when exposed to the parasite populations from Burkina Faso or Cameroon.

**Table 2:** Differences in infection prevalence and intensity between sympatric and allopatric vector parasite couples.

	Oocyst prevalence				Oocyst intensity		
Parasite population	sympatric proportion infected*	allopatric proportion infected*	Odds ratio of allopatric to sympatric (95% CI)	*p*-value	sympatric mean*	allopatric mean*	% increase in allopatric species combination (*p*-value)
PfCM	0.78	0.70	0.65 (0.51, 0.83)	<0.001	15.60	21.10	26% (<0.001)
PfBF	0.61	0.74	1.78 (1.38, 2.30)	<0.001	9.80	12.60	22% (<0.001)
Combined	0.69	0.71	1.06 (0.88, 1.26)	0.53	11.90	15.80	25% (<0.001)

*Note that results between PfCM and PfBF cannot be directly compared as mosquitoes were fed on hosts with different infectivities.

### Differences in oocyst intensity

**Figure 1:**
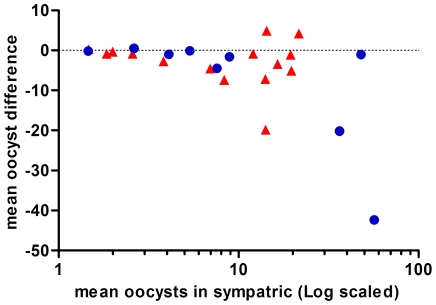
A Bland-Altman plot showing the differences in oocyst intensity between sympatrically and allopatrically infected mosquitoes. Arithmetic means are plotted for PfCM infections as blue circles and PfBF infections as red triangles. When points fall below 0 on the y axis, the mean oocysts in the sympatric infection was lower than for the corresponding allopatric infection.

The difference in oocyst intensity (in infected mosquitoes only) in sympatric vs. allopatric parasite-vector combinations is shown graphically in Figure 1. The Bland-Altman plot shows the difference in mean oocysts between the sympatric and corresponding allopatric infections (mean oocysts in sympatric – mean oocysts in allopatric), plotted against the mean oocysts in the sympatric infection. All points that fall below zero on the y axis represent infections where sympatric couples produced fewer oocysts than the allopatrically infected counterpart. Of a total of 9 successful infections with PfCM and 15 successful infections with PfBF, 8 and 12 of the points respectively fall below zero, meaning that the majority of sympatric infections harbored fewer oocysts. Allowing the infectivity of each host to vary, paired negative binomial regression indicates that sympatric mosquito populations had, on average, 25% fewer oocysts than allopatric populations (*p*<0.001, see Table 2) (i.e. mean oocysts for sympatric populations = 11.9 compared to 15.5 for allopatric populations). Consistent results were seen analyzing the results for each parasite population separately, i.e. significantly fewer PfBF oocysts were found within the sympatric AgBF mosquitoes than the allopatric AgCM mosquitoes and vice versa for PfCM: significantly fewer oocysts were found within the sympatric AgCM mosquitoes than the allopatric AgBF.

## Discussion

This is, to our knowledge, the first study to look for differences in parasite development between populations of wild *P. falciparum* and its natural vector *A. gambiae* in Africa. We present data showing significant differences in infection success between sympatric and allopatric vector-parasite combinations. In terms of infection intensity fewer *P. falciparum* oocysts developed within sympatric *A. gambiae* strains, with no significant differences in prevalence.

The cause of the observed reduced infection intensity in sympatric combinations may be explained by the impact of the parasite on the insect vector. The impact that the malaria parasite has on the life-expectancy of infected mosquitoes is still not clear, likely depending on parasite-mosquito species combinations (reviewed in [5]) and environmental conditions [36]. However, recent evidence from a laboratory model indicates that vector mortality may increase the more gametocytes the mosquito ingests [37]. In regards to the positive relationship between gametocyte density and infection load [38]–[40], these results would imply that infection induced mortality in mosquitoes depends on the number of parasites that infect them. In this study sympatric mosquitoes harbor fewer parasites than their allopatrically infected counterparts and so are expected to be fitter. These results therefore suggest that the *A. gambiae* populations used are locally adapted to the parasite. Interestingly, no effect is seen on infection prevalence between the local vs. foreign species combinations. It is typically assumed that the mosquito will always be fitter when it is not infected (examples of fitness costs of infection and references therein [5], [37], [41]). On this assumption, the results presented here indicate that there is no vector local adaptation for infection prevalence. However, the conclusions drawn on prevalence may not show the whole picture as infections were conducted with blood from gametocyte carriers with 20+gametocytes/µl blood. The effect of increasing gametocyte density on oocyst prevalence has been shown to plateau [40] and indeed in the current data set this is observed at around 20+gametocytes/µl blood (data not shown), therefore increasing the gametocyte density is not expected to affect the infection rate. It therefore cannot be ruled out that different conclusions may be drawn when using lower gametocyte densities. On the other hand the parasite maintains its infection prevalence (at infections with 20+gametocytes/µl) which is crucial for its survival, while reducing infection intensity in local vectors. If these lower intensities reduce the potential parasite induced vector mortality, then it could actually increase the chances of transmission, which would mean that the parasite is locally adapted to the mosquito in parallel.

In the current study we look for differences in infection between 2 *P. falciparum* and 2 *A. gambiae* populations infected sympatrically and allopatrically. One limitation is the use of mosquito colonies which takes it a step away from the situation in nature as colonies show reduced genetic diversity compared to wild mosquitoes [42]. Recently established colonies have been used to minimize this effect but natural polymorphisms involved in adaptive processes may already have been lost from the mosquito genome. Differences imposed due to the number of colonization events also cannot be ruled out as local mosquitoes are colonized once, while distant mosquitoes are colonized twice, once in the local site and a second time in the distant site. The major selection event taking place is to insectary conditions, which are uniform in both sites, however the second colonization could affect fitness due to a further reduction in genetic diversity or inevitable differences in insectary conditions. In this study wing length was used as an indication of fitness [31], [43] and was found not to differ between the local and distant mosquito colonies. This indicates (even if not exhaustively proving) that allopatric mosquito colonies do not suffer any significant reduction in fitness when compared to the sympatric mosquito colonies. The experimental design however allows large numbers of mosquitoes to be experimentally infected at one time, greatly increasing the power to detect differences in *P. falciparum* infection. Two factors that play a major role in determining whether a population is capable of local adaptation are gene flow [44] and genetic variation [33] where reduced within population variability limits local adaptation [45] and high gene flow counteracts effects of selection and local adaptation [23]. In terms of the mosquito, population differentiation is known to be more prominent. Within *A. gambiae* M form, the different patterns of chromosomal inversions are a good example of this. Different inversion patterns are associated with environmental adaptation making it likely that the local environment infers diversity rather than geographic distance [46]–[51]. The two mosquito populations used in this study have different inversion patterns, AgCM has the Forest pattern which is fixed for the standard chromosome arrangement and AgBF has the Mopti pattern which is almost fixed for the 2Rbcu and 2La inversions [52]. Divergence between the Mopti M form and Forest M form is high (Fst = 0.0406, AMOVA = 3.9% of covariance between populations), suggesting reproductive isolation [53]. These differences in the mosquito genetic background, along with different external environments and malaria transmission patterns (see methods) likely presents differing emphases on the selection pressures that determine vector-parasite interactions in the two populations used. In the parasite, merozoite surface protein genes are known to be highly genetically diverse and under selection [54]–[55]. *Plasmodium* genotyping of two MSP loci shows that the two populations are highly genetically diverse with the majority of genetic variability existing within population, with low population divergence between the PfBF and PfCM samples. This is consistent with other studies in Africa where malaria transmission is high (as estimated here by the proportion of mixed infections) [56]–[59]. It has been proposed that out-crossing is responsible for this as the likelihood of a mosquito ingesting a mixed infection is high and in turn that distinct gametes combine in the mosquito midgut [59]–[61]. The low yet significant divergence between the two populations for the MSP2 locus suggests that the two parasite populations are being differentially selected in CM and BF to the extent where gene flow between the two populations is not enough to completely homogenize and counteract the effects of differential selection [23]. This along with the infection phenotype data suggests that each mosquito population is locally adapted to its sympatric parasite population. To our knowledge only one other study exists that compares infection of local (determined to the scale of the country of origin) and geographically distant *P. falciparum* and *Anopheles*. Warren and Collins completed experimental infections with several different mosquito and parasite strains/species. Local and distant infections with *P. falciparum* can be compared for South East Asian and Central American *Anopheles* strains/species. *P. falciparum* was generally more successful in geographically distant *Anopheles*, although the relationship was not always clear [62]. This is consistent with our findings and the lack of clarity could be due to the fact that geographical distance between the mosquito and parasite is not the only determinant that can be linked to infection success. Genetic population divergence is a much better indicator of whether adaptation could be expected and is nicely demonstrated in Joy *et al* [16].

The impact of vector local adaptation in the *A. gambiae*-*P. falciparum* system could be considerable. We predict that genetic distinctions exist between the two parasite populations specifically involved in host-parasite interactions, driven by host-parasite co-evolution. Further studies are underway to compare gene expression profiles of sympatrically infected and allopatrically infected mosquitoes to help identify which genes distinguish these locally adapted phenotypes and are potentially undergoing selection. Determining how genetically diverse those interactions are between different co-evolving (and potentially locally adapted) populations will be crucial for understanding how to interpret research up to now, which rarely stretches to natural populations and even more rarely to multiple sympatric populations. The likely dynamic nature of vector-parasite interactions could mean that much of the current research that does not take this into account may have been misinterpreted [25]. This study reiterates what has previously been suggested that researching vector-parasite interactions in unnatural couples, which could be due to using lab strains, artificial species combinations or simply geographically isolated vectors and parasites, molecular interactions uncovered could differ drastically to those found in nature [7], [63]–[64]. On top of that, on uncovering a molecular basis to, for example, mosquito resistance to malaria, the magnitude of its effect cannot be determined until it is tested in multiple sympatric couples. Locally adapted populations are likely to evolve at different rates and involve different molecular factors [65] and so an important immunity gene (or polymorphism within it) in one mosquito population may not have evolved to play a crucial role in another population.

The results presented in this paper provide the first evidence to suggest local adaptation in the medically important *A. gambiae* – *P. falciparum* system. It highlights that research into vector-parasite interactions and in particular the mosquito immune response to malaria must use multiple natural sympatric couples if results are to be meaningful in uncovering novel malaria control measures. The system should not be over-simplified and considered as a single interacting unit, but rather a very dynamic one varying over space and/or time. More research on the extent of adaptation and specific factors involved is needed to give us a better understanding of the dynamics of malaria transmission, of immune evolution with its potential mosaic effect and future malaria control strategies.

## Materials and Methods

### Ethical Statement

Ethical approval was obtained from the Cameroonian and Burkina Faso National Ethics Committees. All human volunteers were enrolled after written informed consent from the participant and/or their legal guardians.

### Study Sites

Two study sites have been used, one in Cameroon, Central Africa and one in Burkina Faso, West Africa. In Cameroon both mosquitoes and blood donors are from the vicinity of Yaoundé, a rainforest area, where the intensity of malaria transmission is relatively constant throughout the year, but slightly higher during the rainy seasons [66]. In Burkina Faso mosquitoes and blood donors are from the Vallée du Kou, a rice growing area in a savannah region, where malaria transmission is more seasonal [67].

### Mosquito Colonies

*A. gambiae* colonies were established in each site, named here “AgCM” (*A. gambiae* Cameroon, started in January 2006) and “AgBF” (*A. gambiae* Burkina Faso, started in January 2008). Both the M and S *A. gambiae* molecular forms are present in the study sites [51], [68]–[69]. Here we have focused on the M form which is fixed for the Forest pattern of chromosomal inversions in AgCM and Mopti pattern in AgBF [52], which are indicative of differential ecological adaptation [46], [70]. In each study site, one M form colony was established from breeding sites containing only M form larvae or wild caught females that laid eggs individually before species determination by PCR-RFLP [71]. Colonies were maintained in the insectary (12 h day/night cycle, 28 +/− 2°C, 85 +/−5% humidity, with 8% sucrose) and were regularly tested thereafter for pure M molecular form [71]. Once established, eggs from each colony were transferred to the second site (AgCM strain from Cameroon was sent to Burkina Faso and AgBF strain from Burkina Faso to Cameroon) and colonized giving both mosquito strains in both sites.

### Experimental infections

This protocol has been approved by the national and institutional ethical committees. Experimental infections were carried out at each study site infecting both mosquito strains in parallel with local wild *P. falciparum* gametocyte isolates. Mosquitoes therefore fit into one of 4 infection groups: AgCM-PfCM*, AgBF-PfCM, AgBF-PfBF* and AgCM-PfBF (Ag = *A. gambiae*, Pf = *P. falciparum*, CM = Cameroon, BF = Burkina Faso, sympatric combinations are marked with a *). To do this asymptomatic *P. falciparum* gametocyte carriers were selected by producing thick blood smears from school children aged between 5 and 11 from within the study sites. Malaria positive individuals were treated according to national recommendations. A venous blood sample of up to 8 ml was taken from selected carriers with at least 20 gametocytes/µl of blood (considering an average of 8000 white blood cells/µl). In order to limit the potential effect of human transmission blocking immunity [72], the blood was first centrifuged at 2000 rpm at 37°C for 3 minutes and the serum changed to European naive AB serum with 0.225 UI heparin/ml. 500 µl of reconstituted blood was added to 3 membrane feeders/mosquito strain maintained by water jackets at 37°C. Two to three day old female mosquitoes were allowed to feed for up to 30 minutes through Parafilm, un/partially fed mosquitoes were later removed. Fully fed mosquitoes were maintained under standard conditions on an 8% sucrose diet. At day 8 post infection, midguts were dissected in 0.4% mercurochrome stain and viewed under a light microscope where the oocysts can be counted by eye to determine the level of infection in each female. In each field site experimental infections were repeated up to 17 times during August–October 2008.

### Blood meal and wing size differences between mosquito strains

Blood meal and wing size differences were compared between AgCM and AgBF mosquito strains in the Burkina Faso field site. Pools of approximately 60 females of each colony were fed simultaneously by membrane feeding on human blood. As described for the experimental infections, unfed and partially fed mosquitoes were visually sorted and removed. The amount of hematin, a by-product of the decomposition of hemoglobin, was measured for each female [73]. To do this, mosquitoes were placed individually in 30 ml plastic tubes directly after feeding and kept for 3 days to allow complete digestion and all hematin to be excreted. Fecal material of each mosquito was eluted in 1 ml of 1% LiCO_3_ solution and the absorbance of the resulting solution read at 387 nm, using LiCO_3_ solution as a blank, and compared with a standard curve made with porcine serum hematin (Sigma-Aldrich). Three replicates were carried out. Difference in blood meal sizes between strains was tested using the Mann-Whitney test. Wings were removed and mounted on slides for AgCM and AgVK females. Wing sizes were measured as described previously [74] and sizes compared again using the Mann-Whitney test.

### Parasite population differentiation

DNA was extracted from aliquots of *P. falciparum* positive blood samples taken from individuals within each study site between April 2006 and October 2008, including those used in each experimental infection, using DNAzol (Medical Research Centre). Parasites were genotyped for two MSP alleles: MSP1 K and MSP2 FC as previously described [75] measuring nested PCR fragment lengths. Here, we used fluorescently labeled reverse primers and detected sizes on an Applied Biosystems 3130×l Sequencer. Genetic variability and AMOVA were calculated in Arlequin v.3.1 [76] for each marker to measure within population genetic variability and inter population divergence. The proportion of mixed infections was estimated based on the proportion of blood isolates containing more than one allele for either marker. High within population diversity justified treating each infection as an independent observation in the following statistical analyses.

### Statistical Analysis

Hosts vary in their underlying infectivity. In each field site mosquitoes from both strains were fed on the same parasite isolates. This allows the infectivity of allopatric and sympatric parasite-vector combinations to be compared directly using generalized linear mixed models. Microscopy estimates of gametocyte density will not fully explain host infectivity as gametocytes will differ in their viability. The infectivity of each host is therefore allowed to vary at random enabling a paired comparison between sympatric and allopatric species (the fixed effect). The likelihood ratio test was used to determine whether including sympatry information significantly improved the fit of the model. Results are initially shown for the different parasite strains separately before the data is collated and used to see if any trends are consistent across the two field sites. Since a relatively small number of hosts were sampled at each site the infectivity of the two different parasite strains cannot be directly compared. The proportion of mosquitoes developing oocysts (the prevalence of infection) was investigated using hierarchical logistic regression and the results shown as odds ratios. The number of oocysts successfully developing within mosquitoes feeding on the same host tend to be highly aggregated (i.e. overdispersed in relation to the Poisson or random distribution) [77]. As a result hierarchical zero truncated negative-binomial regression models were used to compare the number of oocysts in infected mosquitoes. The arithmetic mean is used to compare the intensity of infection in infected hosts. Substantial and comparable parasite overdispersion was observed in the sympatric (*k* value of the negative binomial distribution = 0.86) and allopatric (*k* value = 0.82) infections. All statistical analyses were completed in R v.2.10.0 [78].

Comparing the infectiousness of different *P. falciparum* strains between Cameroon (PfCM) and Burkina Faso (PfBF) is beyond the scope of this paper due to the difficulty in disentangling the differences in mosquito infectivity due to the biology of the parasite or due to the field site.
